# Evaluation of secondary metabolites of herbal plant extracts as an antiviral effect on infectious bursal disease virus isolates in embryonated chicken eggs

**DOI:** 10.14202/vetworld.2021.2971-2978

**Published:** 2021-11-25

**Authors:** Rawaa Saladdin Jumaa, Dhuha Ismael Abdulmajeed, Abdulkarim Jafar Karim

**Affiliations:** 1 Department of Microbiology, College of Veterinary Medicine, University of Baghdad, Baghdad, Iraq; 2 Unit of Zoonotic Diseases, College of Veterinary Medicine, University of Baghdad, Baghdad, Iraq

**Keywords:** chick embryo, herbal plant, infectious bursal disease virus, Iraq

## Abstract

**Background and Aim:**

Infectious bursal disease attacks the poultry industry, mainly young chickens, causing immunosuppression, and death with high economic losses. This study aimed to evaluate the effects of the monoextract, diextracts, and triextracts of *Quercus infectoria* (QI), *Citrus aurantifolia* (CiA), and *Coffea arabica* (CoA) on infectious bursal disease virus (IBDV) in embryonated chicken eggs (ECEs).

**Materials and Methods:**

The experimental design consisted of three sets of ECEs at 11 days of age, and each set included seven groups (G1-G7). The extracts of QI, CiA, and CoA were inoculated to ECEs by the chorioallantoic membrane method before, in concomitant (mixed) with, and after IBDV infection to the first, second, and third sets, respectively. The monoextract, diextracts, and triextracts of QI, CiA, and CoA were given at 1%, 2%, 5%, and 10% concentrations to G1-G3, G4-G6, and G7, respectively. Real-time polymerase chain reaction identified and confirmed the virus in accordance with the pathological changes.

**Results:**

The monoextract (5-10% concentrations) inhibited IBDV and had no effect on viral infection preinoculation, whereas the monoextract (10% concentration) inhibited IBDV during mixed inoculation and post-inoculation. Diextracts (2-10% concentrations) inhibited IBDV and had no effect on viral infection preinoculation, whereas diextracts (5-10% concentrations) inhibited IBDV during mixed inoculation and post-inoculation. Triextracts (1%, 2%, 5%, and 10% concentrations) inhibited IBDV by ameliorating the pathological changes of the virus and preventing the death of ECEs.

**Conclusion:**

The inoculation of herbal extracts, particularly triextracts, alleviates the pathological changes in ECEs infected with IBDV. This study recommends the oral route in evaluating plant extracts against IBDV in poultry.

## Introduction

Infectious bursal disease (IBD) is caused by a virus that belongs to the Birnaviridae family, called the IBD virus (IBDV). The disease attacks the poultry industry, mainly the bursa of Fabricius of young chickens, causing immunosuppression, vaccine failure, secondary infections, and death with high economic losses [1-3]. IBDV is incriminated in the destruction of the bursa of Fabricius, resulting in the lack of B lymphocytes, which is responsible for humoral immunity [4]. Therefore, immunosuppression and weak immunity make chickens prone to various diseases and vaccination failure [5]. Immunomodulation is a major concern in poultry farms, and innovative products at reasonable rates that can help improve or avoid such diseases are critical for the poultry industry as well as humans [6,7].

Herbal medicine has a promising value at present. It is frequently used in treating many diseases, including incurable ones [8-12]. Such herbal medicines include those derived from *Quercus infectoria* (QI), *Citrus aurantifolia* (CiA), and *Coffea arabica* (CoA). Oak galls (QI), such as many other herbs, have many bioactivities, such as antiviral, antioxidant, antifungal, antibacterial, larvicidal, anti-inflammatory, antiamoebic, antidiabetic, antivenin, and wound healing [13-15]. QI extract has long been used to treat a variety of ailments and for health promotion [13]. Dried lime (CiA) is high in Vitamin C with antioxidant, antimicrobial, diuretic, detoxifying, and anthelminthic properties [16-20] and is used to strengthen the stomach, intestinal, and heart muscles and prevent osteoporosis. These effects are aided by the presence of calcium and riboflavin in the diet. CiA is added as traditional seasoning to meals and taken as herbal tea to treat certain diseases in the Middle East [21]. Coffee (CoA) is among the most widely consumed drinks on the planet, accounting for roughly one-third of the global consumption [22]. It is used as an adsorbent for toxic metals, bioactive composites, and pharmaceutical and cosmetic industries [23-27]. The antioxidant and antimicrobial properties of coffee are attributed to its high polyphenol, flavonoid, dietary fiber, caffeine, and chlorogenic acid content [25,28,29].

Evaluating the efficacy of a purified herbal extract is usually studied using an antioxidant assay, an oxygen radical absorbance ability assay, or a free radical method [30-35]. Moreover, these extracts have antiviral properties that can be enhanced synergistically by combining them with other supplements. Poultry farms have commonly used natural medicinal products as feed supplements with immune-stimulant and stress-relieving properties [36-42].

This study aimed to evaluate the effects of QI, CiA, and CoA extracts in ameliorating the pathological changes associated with IBDV infection.

## Materials and Methods

### Ethical approval

All procedures followed the guidelines of the Animal Care and Use Committee and approved under no. ACUC/2517 on 25 May 2020.

### Study period and location

The study was conducted from September 15, 2020 to January 21, 2021. The study was carried at the Laboratory of Virology, Department of Microbiology, College of Veterinary Medicine, University of Baghdad.

### Sample collection

Twenty samples (bursa of Fabricius) were collected from chickens suspected of having Gumboro disease (IBD) in various areas of Iraq’s Baghdad government. Bursa of Fabricius samples were prepared according to Mutinda *et al*. [43] and stored in 2 mL Eppendorf tubes at −20°C until use.

### Viral detection using real-time polymerase chain reaction (RT-PCR)

RNA bursal cells were isolated and purified using a kit from Qiagen. Viral amplification was carried out by RT-PCR using kits from Genekam Biotechnology AG. Primers (short nucleic acid sequence used in PCR) were used to perform a molecular diagnosis of RNA derived from samples suspected of being infected with IBDV.

### Viral propagation in embryonated chicken eggs (ECEs) through chorioallantoic membrane (CAM)

The prepared samples were inoculated on CAM according to Mutinda *et al*. [43].

### Determination of 50% embryonated lethal dose (ELD50) of IBDV

Virus titer was determined by the pathological changes of IBDV in ECEs and expressed as ELD_50_ per milliliter using the formula of Reed and Muench [44].

### Preparation of plant extracts

Each plant was extracted using 70% (1:5, w/v) methanol according to El-Rabey *et al*. [45].

### Detection of phytochemical agents

The detection of the phytochemical agents of each extract was carried out according to Harborne [46].

### Preparation of stock solution of QI, CiA, and CoA extracts

Ten grams each of QI, CiA, and CoA in 10 mL sterilized distilled water were used to make a stock solution for each extract. The toxicity of each extract was measured using ECEs at various concentrations (1%, 2%, 5%, and 10%).

### Study design

The study design consisted of three sets of treatments. These sets were infected with IBDV and treated with plant extracts at 1 h before, 0 time, and 1 h after viral inoculation (Table-1). For each set, seven groups of chick embryos, five each, were treated as follows: Monoextract QI, monoextract CiA, monoextract CoA, diextracts QI and CiA, diextracts QI and CoA, diextracts CiA and CoA, and triextracts QI, CiA, and CoA. The effect was observed for 5 days after inoculation.

**Table-1:** Design of the study.

Groups	G1 (QI)	G2 (CiA)	G3 (CA)	G4 (QI+CiA)	G5 (QI+CA)	G6 (CiA+CA)	G7 (QI+CiA+CA)
		
	Mono extract			Di extract			Tri extract
	1. 1% of extract						
Sub-groups	2. 2% of extract						
(Treatments)	3. 5% of extract						
	4. 10% of extract						
	5. DW (−ve control)						
	6. IBDV (+ve control)						
Timing	*Pre-viral inoculation (1^st^ set)			Zero time-viral inoculation (2^nd^ set)		*Post- viral inoculation (3^rd^ set)	
Parameters	1. RT-PCR						
	2. Pathological changes.						

QI=*Quercus infectoria*, CiA=*Citrus aurantifolia*, CoA=*Coffea arabica*, DW=Distilled water, IBDV=Infectious bursal disease virus

* (1 h). RT-PCR=Real-time polymerase chain reaction

### Toxicity test

According to Eladl *et al*. [5], the acute toxicity for each extract (monoextract, diextracts, and triextracts) was determined as LD_50_ in ECEs. In a nutshell, 12 (11-day-old) ECEs were inoculated with three doses of 1, 10, and 100 g/mL of each plant extract (monoextract, diextracts, and triextracts)/distilled water, with one group of ECEs serving as the untreated control group. Toxicity symptoms include the death of ECEs after 24 h. Every plant extract (monoextract, diextracts, and triextracts) showed no signs of toxicity or mortality in ECEs according to the toxicity test.

### RT-PCR for harvested CAM in treated ECEs

Harvested virus (CAM) from treated and infected ECEs was tested using RT-PCR.

## Results

### Methanolic extracts of QI, CiA, and CoA

According to Banso and Adeyemo [47], the methanolic extracts of QI, CiA, and CoA produced dark-brown crystals, dark-brown sticky oil, and golden yellow crystals at 64%, 42%, and 19%, respectively.

### Phytochemical agents

The phytochemical analysis of each methanolic extract revealed the existence of all secondary metabolic agents, except for resin, steroid, and saponin, as shown in Table-2.

**Table-2:** Phytochemical analysis of *Quercus infectoria* (QI), *Citrus aurantifolia* (CiA) and *Coffea arabica* (CoA).

Phytochemicals (Active components)	QI	CiA	CoA
Tannin	+	+	+
Phenol	+	+	+
Saponin	+	+	-
Flavonoid	+	+	+
Steroid	+	−	−
Terpenoid	+	+	+
Glycoside	+	+	+
Resin	−	+	−
Alkaloid	+	+	+

+=Present, −=Not present.

### Toxicity of extracts

According to the toxicity test, no signs of toxicity or mortality in ECE occurred within 24 h in all plant extracts.

### IBDV diagnosis

**Figure-1:**
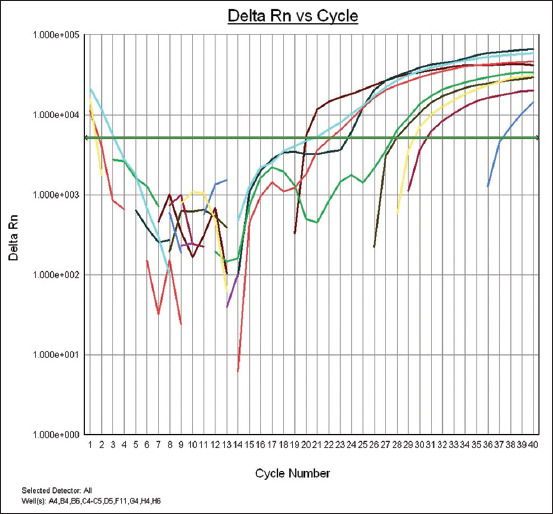
A plot of amplification of the viral protein 2 gene by real-time polymerase chain reaction for detection of infectious bursal disease virus revealed positive bursa of Fabricius samples.

Of 20 samples, only eight bursa of Fabricius samples were confirmed positive for IBDV using RT-PCR (Figure-1).

### Pathological changes

**Figure-2:**
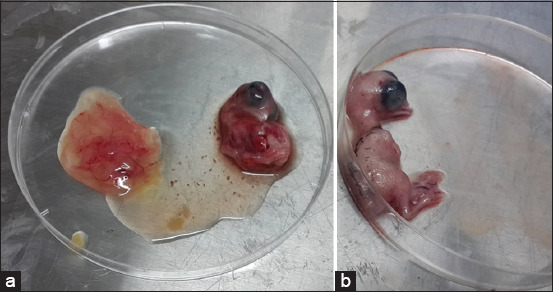
Infectious bursal disease virus-infected embryonated chicken eggs display pathological changes. (a) Infected (b) control.

Positive samples were used to inoculate ECEs using RT-PCR. Three days after infection, pathological changes were observed. The first passage revealed no changes in the embryo and monitored ECEs, but the second passage revealed death and dwarfing of the infected embryo and hemorrhage on the infected embryo’s body (Figure-2a). In contrast, the control showed no changes (Figure-2b).

### Titration of isolated IBDV in ECEs (ELD_50_)

In ECEs, the second passage titer for the first isolated IBDV was 10^3^/100 µL, whereas the second passage titer for the second isolated IBDV was 10^2.5^/100 µL. The effect of herbal plant extracts on ECEs infected with IBDV is explained in Table-3.

**Table-3:** Effect of secondary metabolic agents in ameliorating pathological changes of IBDV of pre-, mixed-, and post-treated extract.

Time	Treatments	G1 (QI)	G2 (CiA)	G3 (CoA)	G4 (QI and CiA)	G5 (QI and CoA)	G6 (CiA and CoA)	G7 (QI & CiA and CoA)
Pre-treated	DW	─ve	─ve	─ve	─ve	─ve	─ve	─ve
	IBDV	DP	DP	DP	DP	DP	DP	DP
	1% extract	D48*	D48*	D48*	D72*	D72*	D72*	─ve
	2% extract	D72*	D72*	D72*	─ve	─ve	─ve	─ve
	5% extract	─ve	─ve	─ve	─ve	─ve	─ve	─ve
	10% extract	─ve	─ve	─ve	─ve	─ve	─ve	─ve
Mixed-Treated	DW	─ve	─ve	─ve	─ve	─ve	─ve	─ve
	IBDV	DP	DP	DP	DP	DP	DP	DP
	1% extract	D48*	D48*	D48*	D72*	D72*	D72*	─ve
	2% extract	D72*	D72*	D72*	D98*	D98*	D98*	─ve
	5% extract	D98*	D98*	D98*	─ve	─ve	─ve	─ve
	10% extract	─ve	─ve	─ve	─ve	─ve	─ve	─ve
Post-Treated	DW	─ve	─ve	─ve	─ve	─ve	─ve	─ve
	IBDV	DP	DP	DP	DP	DP	DP	DP
	1% extract	D48*	D48*	D48*	D72*	D72*	D72*	─ve
	2% extract	D72*	D72*	D72*	D98*	D98*	D98*	─ve
	5% extract	D98*	D98*	D98*	─ve	─ve	─ve	─ve
	10% extract	─ve	─ve	─ve	─ve	─ve	─ve	─ve

DW=Distilled water, IBDV=Infectious bursal disease virus, −ve=No death, D=Death, *P=*Pathological changes, DP=Death and pathological changes,

* death time (hours). QI=*Quercus infectoria,* CiA=*Citrus aurantifolia*, CoA: *Coffea arabica*

### RT-PCR for harvested CAM in treated ECEs with herbal plants

**Figure-3:**
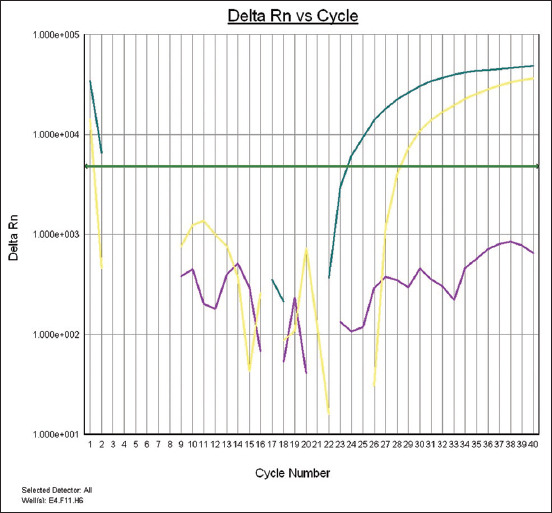
Plot of amplification RT-real time PCR for viral protein 2 gene in harvested chorioallantoic membrane. The green line refers to the 50% triextract group, yellow line refers to positive control, and the violet line as negative control.

RT-PCR effectively detected IBDV inoculated on CAM with triextracts at 50% concentration, as shown in Figure-3.

## Discussion

The effects of three plants, alone (monoextract) or mixed (diextracts and triextracts), on ECEs infected with IBDV were investigated in this study. RT-PCR was used to detect viral protein 2 (VP2), the spike of the very virulent strain of IBDV (vvIBDV). This is in line with the findings of Jumaa *et al*. [48], who detected the Iraqi strain of IBDV using RT-PCR and isolated it using fibroblasts of ECEs. Therefore, these results agreed with Cheggag *et al*. [49], who reported that this technique could detect highly virulent IBDV strains from classic and variant IBDV strains. These results also agree with Jumaa *et al*. [50], who confirmed the VP2 of Iraqi vvIBDV strain using sequence analysis.

In this study, plant extracts were inoculated on ECEs against IBDV challenge during preincubation, 0 time (mixed), and post-infection (Table-3). The monoextract (5-10% concentrations) inhibited IBDV and had no effect on viral infection preinoculation, whereas the monoextract (10% concentration) inhibited IBDV during mixed and post-inoculation. Diextracts (2%, 5%, and 10% concentrations) inhibited IBDV and had no effect preinoculation, whereas diextracts (5–10% concentrations) inhibited IBDV during mixed inoculation and post-inoculation. Triextracts (1%, 2%, 5%, and 10% concentrations) inhibited IBDV and had no effect on IBDV infection preinoculation, during mixed inoculation, and post-inoculation. These results may be due to the activity of phytochemical agents in herbal extracts, where post-inoculation treatment consistently outperformed preinoculation and mixed treatments.

These findings coincided with many investigations on aqueous and alcoholic extracts against IBDV. Aslam *et al*. [39] revealed a 100% inhibitory effect for methanolic extracts of 11 Cholistani plants and effectiveness in controlling the growth of poultry viruses, such as IBDV and infectious bronchitis, under *in vitro* conditions. Ahmad *et al*. [40] illustrated the ethanolic extracts of different plants, including *Glycyrrhiza glabra*, *Moringa oleifera*, *Phyllanthus emblica*, and *Eugenia jambolana*, against IBDV. Virmani *et al*. [41] reported that aqueous and alcoholic extracts of *M. oleifera*, *Holarrhena antidysenterica*, *Synzium aromaticum*, *Allium sativum*, *Piper nigrum*, and *Azadirachta indica* were effective in controlling IBDV growth. The anti-IBDV activity of root *Withania somnifera* hydroalcoholic extracts was also reported [42].

The secondary metabolites in the QI, CiA, and CoA extracts in this study, including tannin, phenol, saponin, flavonoid, terpenoid, glycoside, resin, and alkaloid, act as bioactive components with effective therapeutic value [51,52]. Furthermore, several reports indicated the phytochemical ingredients of these extracts. Nutrients and herbal medicines are effective antiviral, antioxidant, anticancer, antibacterial, anti-inflammatory, and cardioprotective agents, promote immune response, and are interesting candidates for healthcare and biopharmaceutical applications [53-56]. These phytochemicals use a variety of mechanisms to prevent viral replication and control viral infection. These mechanisms block viral binding to prevent viral attachment sites or host receptors. Other strategies include inhibiting viral replication by attacking the viral enzyme (DNA or RNA polymerase, reverse transcriptase, and protease) or the viral assembly by inhibiting posttranslationally modified viral proteins [57,58].

The inhibitory effect of viral growth in chick embryos is mainly attributed to the phytochemical components (tannin, flavonoid, and phenolic compounds) detected in this study. Cheng *et al*. [59] revealed that tannin, flavonoid, and phenolic compounds are traditional medicines used to treat various health issues, including herpes simplex virus type 2. Furthermore, Ashok and Upadhyaya [60] showed that tannin is used as an astringent, providing a plethora of natural sources for treating inflamed and injured tissues, such as burns, and preventing infections. Perin *et al*. [61] illustrated the phytochemicals used to treat mouth and throat inflammation, gastritis, irritable bowel disorders, and other illnesses. Tannin forms a protective layer on the cellular surface and multiple hydrogen bonds between their phenolic groups and the NH groups of the peptides that cause protein shrinkage. This was supported by Ghildiyal *et al*. [62], who showed that phytochemicals, such as flavonoids, are used as antivirals because of the inhibition of protein phosphorylation that limits viral RNA replication.

Furthermore, several researchers exhibited that tannin has significant biological activities with antiviral activity against a wide range of DNA and RNA viruses. These biological activities attack extracellular virions by inhibiting different steps of viral replication, including viral attachment to cell receptors, viral penetration, viral assembly, and transport proteins, polysaccharides, and viral enzymes [63]. Furthermore, the activity of tannins is mainly attributed to their ability to bind to viral structural or nonstructural proteins (capsid or viral enzymes) necessary for viral replication or structural proteins involved in the creation of new viral particles [64].

The flavonoid, a polyphenolic phytochemical component, detected in the extracts in this study has antiviral activity against viruses, particularly segmented RNA viruses [64]. Zakaryan *et al*. [65] described the antiviral activity of baicalein, a flavonoid that inhibits the various steps of viral RNA replication. Another flavonoid, fisetin, was successfully reported to inhibit virus replication *in vitro* [66]. In addition, flavonoids have been linked to the inhibition of intracellular viral replication in the early stages [66,67]. Furthermore, flavonoid antiviral activity is due to the high water solubility of their structure, which has been recognized as metabolites, and small molecules responsible for detoxification and boosted biological effects. Sodium rutin sulfate inhibited viral replication and blocked virus entry into host cells by interrelating with the glycoprotein of the viral envelope. Previous study has suggested that viral RNA polymerase inhibition is possible [64].

Another secondary metabolite detected in this study is terpene, which acts as a viral inhibitor. Terpenes consist of five carbon isoprene units linked by simple hydrocarbons. Terpenoids are simply modified terpenes and have different functional groups at various positions. Terpenoids, also referred to as isoprenoids, are naturally found in plants and have a significant role in herbal treatments with many medical applications [60]. According to Fayyad *et al*. [68], terpenes can disrupt virus attachment to the cellular membrane and inhibit viral DNA replication. Similarly, Yang *et al*. [69] extracted and tested terpenoids for antiviral activity against RNA viruses, and these compounds inhibited viral replication by preventing viral structural protein synthesis and inhibiting genes responsible for coding the viral spike, viral membrane protein, and viral nucleocapsid protein.

However, most components of the secondary metabolites detected in this study include tannins, flavonoids, and phenolic acids that have antioxidant activity and are broadly distributed in several plants, acting as a protective mechanism against oxidative stress. Overproduction of free radicals, and the subsequent emergence of oxidative stress, is a pathogenic factor in various viral infections. Oxidative damage is a multifaceted biochemical state that occurs when oxidative stress on biomolecules increases and the oxidation of nonprotein and protein thiol groups controls the oxidative stability of a cell [69]. Cellular damage caused by viral infection generated through free radical overproduction has been related to >200 clinical illnesses [70]. Many viral infections cause the production of reactive oxygen and nitrogen species linked to epithelial wall dysfunction and lung tissue damage, increasing susceptibility to secondary infections [71]. As a result, antioxidant treatment is an appealing and effective treatment approach for viral infections. The antioxidant properties for several tannin-rich extracts are effective against the development of many RNA or DNA viral infections, improving survival rates while considerably decreasing lipid peroxidation and increasing the oxygen radical absorbance capacity in splenocytes [72,73].

The presence of flavonoids and resins, the phytochemical components detected in this study, indicates the antiviral activity of these components. These results agreed with Pang *et al*. [74], who detected the inhibition of viral replication in the tested resins. The antioxidant activity of resins against viral infections is complemented by intense changes in cell-tissue metabolism, resulting in the severe generation of reactive oxygen species [75]. Alkaloids are also one of the secondary metabolites of these extracts in this study. Some plants from other studies have a nitrogen atom in their ring structure. They are observed at higher plants and have various biological activities, including antiviral, antifungal, antibacterial, anticancer, and antiasthmatic properties. Approximately 43 alkaloids have anti-RNA virus activity. The antiviral activity of alkaloids against RNA viruses may be due to the induction of immune system interferons. Some alkaloids stimulate macrophage activity, allowing them to phagocytose and destroy the virus. At various stages of replication, alkaloids inhibit RNA viral infection. Some of them either inhibit viral protein synthesis or interfere with other stages of replication [76,77].

In addition, the phytochemicals detected in this study, such as tannins, glycosides, and saponins, may have immunomodulatory properties. Tannins have immunomodulatory effects, with various substances exhibiting various mechanisms of action that improve macrophage functionality or induce the excretion of cytokines, including interleukin (IL)-1, IL-2, and tumor necrosis factor [63]. Guo *et al*. [78] found that glycosides have the ability to inhibit viral replication and enhance the innate immune system against RNA viruses. Saponins have been found in a variety of higher plants and have a broad range of pharmacological activities, for example, anti-inflammatory, vasoprotective, gastroprotective, and immunomodulatory effects. Saponins stimulate the components of a specific immunity as well as monocyte proliferation. Saponin, either as a crude mixture or as a purified compound, has increased immune-cell proliferation and antibody production *in vitro* [79].

## Conclusion

The inoculation of herbal extracts, particularly triextracts, alleviates the pathological changes in ECEs infected with IBDV. This study recommends the oral route in evaluating QI, CiA, and CoA extracts against IBDV in chickens.

## Authors’ Contributions

RSJ: Designed the experiment. RSJ and AJK: Interpreted the data and drafted the manuscript. DIA and RSJ: Carried out the experiment, investigation, and resources, DIA, AJK, RSJ. RSJ, and AJK: Performed the statistical analysis and edited the manuscript. All authors have read and agreed to the published version of the manuscript.
